# Personality traits and disorders in Alzheimer's disease

**DOI:** 10.1002/brb3.2938

**Published:** 2023-03-14

**Authors:** Joana Henriques‐Calado

**Affiliations:** ^1^ Faculdade de Psicologia Universidade de Lisboa, Alameda da Universidade Lisboa Portugal; ^2^ CICPSI, Faculdade de Psicologia Universidade de Lisboa, Alameda da Universidade Lisboa Portugal

**Keywords:** aging, Alzheimer's disease, personality, psychopathology, traits

## Abstract

**Background:**

The relationships between axis II personality disorders and the normative personality traits were explored in the context of current and pre‐morbid personality assessment in Alzheimer's disease (AD).

**Methods:**

The study was conducted with four groups who were administered the NEO‐FFI and the PDQ‐4+, in the form of individual interview sessions. Current personality measure: consisting of 44 female participants (AD group) and, the control group, consisting of 80 female participants from the population at large. Pre‐morbid personality measure: AD group informants (*n* = 40); control group informants (*n* = 42).

**Results:**

The results are in line with the literature review and provide new research data. By factorial discriminant analysis, the current and pre‐morbid personality variables that differentiate AD from control groups are identified. The personality traits variables are the best discriminators such as low agreeableness, low openness to experience, and high neuroticism, suggesting that the maladaptive personality functioning can be described extending the range of psychopathology to a dimensional approach.

**Conclusions:**

The study of personality variables seems to suggest, in their inclusion, the possibility to increase sensitivity toward an assessment in AD.

## 1 INTRODUCTION

People with Alzheimer's disease (AD) commonly exhibit changes in personality, along with behavioral and psychological symptoms, that sometimes precede the other early clinical manifestations of the condition, such as cognitive impairment and mood changes (Caselli, 2015; Cipriani et al., 2015; Pocnet et al., 2011, 2013, 2012; Wahlin & Byrne, 2011). Novel findings have been enhancing the understanding of the simultaneous association between personality traits and cognitive status and death, as well as cognitive health span and longevity (Yoneda et al., 2022). Personality changes in AD have been highlighted in the literature and may be a potential early clinical indicator (Duberstein et al., 2010; Duchek et al., 2007; Henriques‐Calado et al., 2016, 2017, 2018; Pocnet et al., 2011, 2013, 2012; von Gunten et al., 2009; Wahlin & Byrne, 2011). Some researchers suggest that the pre‐morbid characteristics of personality may represent a risk factor for AD, and thus, pre‐morbid personality should differ between patients and controls (Balsis et al., 2005; Duberstein et al., 2010; von Gunten et al., 2009). The advances in knowledge about the association between personality and neuropathology AD have come to be highlighted in the literature (Gahr et al., 2012; Tautvydaitė et al., 2017; Terracciano et al., 2022, 2013). Multiple research data have shown that some pre‐morbid personality characteristics even play a role in modifying the disease process or its phenotypic expression (Gilbert & Herbst, 2014; von Gunten et al., 2009). Otherwise, research into current and premorbid personality traits or disorders as early markers of AD has been neglected (Duberstein et al., 2010; Pocnet et al., 2011; von Gunten et al., 2009). Besides, the study of personality disorders appears as relevant in understanding these personality changes in Dementia, while remaining unusual (Kunik et al., 1994; Gilbert & Herbst, 2014; Holwerda et al., 2012; Mordekar & Spence, 2008; Nicholas et al., 2010; Segal et al., 2006; Trull & Widiger, 2003). The assessment of personality in AD should, in the future, be included in its diagnosis, since these data have implications for the prevention, treatment of symptoms and for the etiological knowledge of these diseases (Balsis et al., 2005; Duchek et al., 2007; Duberstein et al., 2010; Terracciano et al., 2013, 2014).

The research on maladaptive personality functioning within an aging population continues to provide compelling support for the contribution of personality to mental health and compared specific traits and personality disorders assigned by the Axis II of DSM, which is of considerable social and clinical significance (Debast et al., 2014; Lynam & Widiger, 2001; Samuel & Widiger, 2004; Schroeder et al., 2002; Schuster et al., 2013; Tackett at al. et al., 2009; Widiger, 2005; Widiger & Seidlitz, 2002). Arguably, the most commonly used model of normal‐range personality traits is the Five‐Factor Model (FFM) (Widiger et al., 2002). There is a considerable amount of research that affords support for the perspective that personality psychopathology can be captured by this general personality dimensions (Bagby et al., 2008; Schroeder et al., 2002; Tackett et al., 2009). The FFM has the potential to provide a valid and scientifically sound framework from which to assess personality psychopathology (Bagby et al., 2008; Widiger, 2005). Almost of research examining personality change and dementia has been retrospective, using informant report (Balsis et al., 2005; Busch et al., 2015). However prospectively measured self‐report of personality, prior to dementia diagnosis, would enhance the literature (Busch et al., 2015; Yoneda et al., 2017), since also the measures of personality change should include trait change; using a self‐report personality trait assessment such as the NEO Personality Inventory which may provide more comprehensive information regarding specific personality trait change (Yoneda et al., 2017). This could be quite useful in developing a more integrative understanding of the processes by which personality dispositions lead to either a resilience or vulnerability to psychopathology (Lynam & Widiger, 2001; Miller et al., 2001; Widiger & Seidlitz, 2002; Saulsman & Page, 2004, 2005; Bagby et al., 2008; Samuel & Widiger, 2008; Schuster et al., 2013; Debast et al., 2014).

### 1.1 Aim of the study

The study sets out to identify the variables of the current and pre‐morbid personality (and abnormal personality) that distinguish AD from the control groups. Based empirically on the study, and from a preventive and personality evaluation perspective, it aims to propose, for future research purposes, a set of personality variables to be considered in the assessment of AD diagnosis, with a view to increasing the sensitivity of an early diagnosis and being of practical use for current clinical diagnosis.

The aim is to address the identification of personality variables in AD, through discriminant analysis methods that differentiate this pathology from aging, with personality evaluation methods, by intersecting the normative personality traits and disorders.

## 2 METHOD

### 2.1 Participants

The Alzheimer´s disease group (AD group) is composed of 44 female, Caucasian participants of Portuguese nationality, resident in an urban environment with a clinical diagnosis of AD (onset), aged 65 years or above (*M*_Age_ = 81.36 years, *SD* = 6.47 years), with an average of 7.61 years of schooling (*SD* = 4.00 years), and an average of 17.59 points (*SD* = 4.44) in the Mini Mental State Examination.

The control group is composed of 80 female, Caucasian participants, from the general population, of Portuguese nationality, resident in an urban environment, aged 65 years or above (*M*_Age_ = 75.84 years, *SD* = 6.12 years), with an average of 8. 94 years of schooling (*SD* = 2.75 years), and an average of 27.81 points (*SD* = 2.08) in the Mini Mental State Examination.

The AD group informants (*n* = 40) and the control group informants (*n* = 42) are the respective relatives, providing assessments of the pre‐morbid personality characteristics.

### 2.2 Measures

#### 2.2.1 Socio‐demographic questionnaire

#### 2.2.2 Mini Mental State Examination (MMSE)

A 30‐point questionnaire with a total score used extensively in clinical and research settings to measure cognitive impairment.

#### 2.2.3 The NEO‐Five Factor Inventory (NEO‐FFI)

The NEO‐FFI (Costa & McCrae, 1992) is a short‐form of the NEO Personality Inventory (NEO PI‐R), which operationalizes the FFM. The NEO‐FFI contains 60 items, and participants are asked to respond on a 5‐point Likert scale ranging from 0 (strongly disagree) to 4 (strongly agree). The NEO‐FFI scales yield scores for the following personality domains (traits): neuroticism, extraversion, openness to experience, agreeableness, and conscientiousness.

#### 2.2.4 The Personality Diagnostic Questionnaire (PDQ−4+)

The PDQ‐4+ is a self‐report questionnaire with 99 items based on true/false answers, designed to generate diagnoses that are compatible with the diagnostic criteria of the DSM‐IV Axis II for personality disorders (Hyler, 1994). The PDQ‐4+ assesses the 10 personality disorders (scales) and respective clusters included in the DSM‐IV and also further two personality disorders (negativistic and depressive), which appear in the DSM‐IV in Appendix B. It allows for a global personality disorder index (PDQ‐4+ Total) (Hyler, 1994).

An informant version was introduced in this study, an adapted version from the NEO‐FFI and PDQ‐4+, created for empirical research purposes. This methodology follows the procedure adopted in other works (e.g., Osborne et al., 2010; Pocnet et al., 2011; von Gunten et al., 2009; Wahlin & Byrne, 2011). With a view to retrospectively evaluating the relative of the Informant, the initial instruction is as follows: “Think of your relative before the age of 60 years. Remember what she was like in the past, throughout her whole life, and answer the following questions”.

### 2.3 Procedure

The present research study was authorized by the Administrative and Clinical Boards of the Institutions. Participants were clarified as to the aims of the study and provided their informed consent. No compensation was given. It complies with Portuguese Psychologists Board ethical standards.

#### 2.3.1 AD group and AD group informants

The AD group sample (Table 1) was mainly collected (± 69%) at a Psychiatric Hospital Center (Psychiatry and Neurology Outpatients) and (±31%) at geriatric centers (Henriques‐Calado et al., 2017, 2018). Inclusion criteria were as follows: female; 65 years or above; clinical diagnosis of AD (onset); absence of psychiatric or neurological co‐morbidity; with intelligibility and understanding capacities and a minimally stable emotional state to collaborate in psychological evaluation tasks and interpersonal relations. It is clarified that the affective disorders or personality disorders were excluded, although there were no screening tools for mood disorders. It should be noted that no biomarkers for the diagnosis of Alzheimer's disease were used. Application of the protocol was conducted in two face‐to‐face individual sessions, by a psychologist trained for such purpose, corresponding to a total period of approximately 2 h (Henriques‐Calado et al., 2017, 2018).

**TABLE 1:** Demographics and descriptive statistics

Variables	AD group, *n* = 44								Control group, *n* = 80								
	*n*	%	*M*	*SD*	*Me*	*Mo*	Min	Max	*n*	%	*M*	*SD*	*Me*	*Mo*	Min	Max	*p*‐value*
Age, years			81.36	6.47	82.00	80.00	65.00	92.00			75.84	6.12	75.00	74.00	65.00	91.00	.001
65–80	16	36.40	74.56	4.84	73.50	80.00	65.00	80.00	63	78.80	73.52	4.42	74.00	74.00	65.00	80.00	
>80	28	63.60	85.27	3.35	84.50	81.00	81.00	92.00	17	21.30	84.41	3.16	83.00	81.00	81.00	91.00	
Schooling, years			7.61	4.00	4.00	8.00	.00	17.00			8.94	2.75	8.00	8.00	.00	17.00	.02
0	6	13.60							2	2.50							
1–4	0	.00							1	1.30							
5–12	35	79.50							73	91.30							
≥ Licentiate degree	3	6.80							4	5.00							
Marital status																	.46
Single	4	9.10							6	7.50							
Married	10	22.70							35	43.80							
Widowed	28	63.60							34	42.50							
Divorced	2	4.50							5	6.30							
With whom she lives																	
Alone	7	15.90							40	50.00							
Husband	7	15.90							35	43.75							
Children	10	22.70							5	6.25							
Family/friends	2	4.50							0	0							
Geriatric center	18	40.90							0	0							
MMSE (Total score)			17.59	4.44	16.00	15.00					27.81	2.08	28.00	29.00			.001

* *t*‐test or chi‐square.

#### 2.3.2 Control group and control group informants

Collection of the control group (Table 1) sample was carried out at a day center (19 participants) and by means of a “snowball” procedure (61 participants). Inclusion criteria were as follows: female; 65 years or above; from the general population; absence of diagnosed or evident psychiatric or neurological disorder; with intelligibility and understanding capacities and a minimally stable emotional state to collaborate in psychological evaluation tasks and interpersonal relations. It is clarified that the affective disorders or personality disorders were excluded, although there were no screening tools for mood disorders. As regards collection from the control group, each informant was always a relative of each participant. The research protocol and its application were conducted as the previously described situation.

### 2.4 Data analysis

Discriminant factor analysis methods are used to identify the current and pre‐morbid personality variables that differentiate AD from the control groups. Discriminant analysis classifies sets of patients or measures into groups based on multiple measures simultaneously and is most simply thought of as regression analysis when the variable to be predicted is binary (Riffenburgh & Gillen, 2020). A line is constructed between the two groups in a way that minimizes misclassifications. Patients whose results appear on one side of this separator are classified as most likely to have arisen from one group and those with results on the other side are classified as likely to be from the other group. In the discriminant analysis, the goal is to maximize the differences among the groups (Riffenburgh & Gillen, 2020). Since discriminant factor analysis is an exploratory technique with no distribution requirements, it enables a serialized identification of the most important predictor variables for the differentiation of groups. Hence, the maximum Mahalanobis distance criterion was considered, with a view to identifying a sub‐set of variables that would guarantee the best discrimination, while adding a new variable to the previous sub‐set at each step. The rates (%) of individuals correctly classified in their groups were estimated in the base sample as the samples were small and the likelihood of the equal groups was considered a priori. These methods are used primarily from an exploratory/explanatory perspective and aim to discover whether personality variables (personality traits and personality disorders), in terms of their current and pre‐morbid evaluations, make it possible to distinguish the groups defined a priori (current personality study in AD: AD group and control group; pre‐morbid personality study in AD: AD group informants and control group Informants) with a view to understanding personality functioning in AD.

The following variables were considered for analysis: the five personality dimensions of the NEO‐FFI (neuroticism, extraversion, openness to experience, agreeableness, conscienciousness); the personality disorder variables of the PDQ‐4+ (PDQ‐4+ total, clusters A, B, C, Appendix B, scales), the socio‐demographic variables (age, schooling), and the clinical variable (MMSE total).

The results presented in Tables 2, 3, 4, 5 provide the variable that was introduced at each step, the rate (%) of estimated well classified individuals in the base sample, and the mean of the respective variable of each group. The maximum limit of steps was based on the previous step to the next step from which point there was a continuous decrease in the rate of well‐classified individuals in the base sample.

**TABLE 2:** Discriminant factor analysis between the Alzheimer´s disease group (AD group) and the control group for current personality study in Alzheimer's disease

			*M* Groups in base sample	
Step	Variables	% Estimated well classified individuals in the base sample	AD group (*n*= 44)	Control group (*n*= 80)
1	Agreeableness (NEO‐FFI)	74.19	26.06	**33.39**
2	Openness (NEO‐FFI)	84.68	15.70	**22.93**
3	Age	82.26	**81.36**	75.84
4	Neuroticism (NEO‐FFI)	84.68	**32.73**	24.68
5	Extraversion (NEO‐FFI)	84.68	22.70	**25.98**

*Note*: The best rates of well classified individuals are underlined; the highest results are displayed in bold.

**TABLE 3:** Discriminant factor analysis between the Alzheimer´s disease group (AD Group) and the control group for current personality study in Alzheimer's disease with introduction of the PDQ‐4+ scales

			*M* Groups in base sample	
Step	Variables	% Estimated well classified individuals in the base sample	AD group (*n*= 44)	Control group (*n*= 80)
1	Agreeableness (NEO‐FFI)	74.19	26.06	**33.39**
2	Openness (NEO‐FFI)	84.68	15.70	**22.93**
3	Age	82.26	**81.36**	75.84
4	Narcissistic (PDQ‐4+)	87.90	**54.50**	12.50
5	Negativistic (PDQ‐4+)	87.10	**43.18**	21.25
6	Dependent (PDQ‐4+)	88.71	**45.50**	10.00
7	Neuroticism (NEO‐FFI)	87.10	**32.73**	24.68
8	Borderline (PDQ‐4+)	89.52	**40.90**	22.50

*Note*: The best rates of well classified individuals are underlined; the highest results are displayed in bold.

**TABLE 4:** Discriminant factor analysis between the Alzheimer´s disease group informants (AD group informants) and the control group informants for current personality study in Alzheimer's disease

			*M* Groups in base sample	
Step	Variables	% Estimated well classified individuals in the base sample	AD group informants (*n*= 40)	Control group informants (*n*= 42)
1	Openness (NEO‐FFI)	65.85	16.03	**23.62**
2	Conscientiousness (NEO‐FFI)	71.95	**38.65**	33.64
3	Agreeableness (NEO‐FFI)	74.39	26.90	**31.43**
4	Cluster C (PDQ‐4+)	80.49	**77.50**	76.20
5	Neuroticism (NEO‐FFI)	75.61	**27.68**	23.50
6	Appendix B (PDQ‐4+)	80.49	**52.50**	28.10

*Note*: The best rates of well classified individuals are underlined; the highest results are displayed in bold.

**TABLE 5:** Discriminant factor analysis between the Alzheimer´s disease group informants (AD group informants) and the control group informants for current personality study in Alzheimer's disease with introduction of the PDQ‐4+ scales

			*M* Groups in base sample	
Step	Variables	% Estimated well classified individuals in the base sample	AD group informants (*n*= 40)	Control group informants (*n*= 42)
1	Openness (NEO‐FFI)	65.85	16.03	**23.62**
2	Schizotypical (PDQ‐4+)	75.61	15.00	**35.70**
3	Agreeableness (NEO‐FFI)	76.83	26.90	**31.43**
4	Conscientiousness (NEO‐FFI)	84.15	**38.65**	33.64
5	Obsessive‐Compulsive (PDQ‐4+)	86.59	**75.00**	69.00
6	Dependent (PDQ‐4+)	84.15	**20.00**	7.10
7	Depressive (PDQ‐4+)	85.37	22.50	**28.57**
8	PDQ‐4+ Total (PDQ‐4+)	85.37	**38.50**	32.50
9	Narcissistic (PDQ‐4+)	90.24	**30.00**	11.90
10	Borderline (PDQ‐4+)	90.24	**35.00**	16.70

*Note*: The best rates of well classified individuals are underlined; the highest results are displayed in bold.

## 3 RESULTS

### 3.1 Current personality study in AD: Discriminant factor analysis between the AD Group and the Control Group

Table 2 presents the results of discriminant factor analysis between the AD group and the control group. As for the current personality characteristics, it is shown that the dimensions agreeableness and openness to experience are those that best distinguish between the AD group and the control group, with the AD group presenting lower results. It should be noted that the percentage of well‐classified individuals by the two variables taken together is of 84.68%.

The results of the introduction of the twelve personality disorder scales of the PDQ‐4+ are presented in Table 3, in addition to the previous, afore‐mentioned variables, and the object of analysis in Table 2. It is shown that the dimensions agreeableness and openness to experience are those that best distinguish between the AD group and the control group, with the AD group presenting lower results. The percentage of well‐classified individuals by the two variables taken together may be observed at 84.68%. Also in the final step, the negativistic, dependent, and borderline scales, and the dimension of neuroticism, are those that best distinguish between the AD group and the control group, with the AD group presenting higher results. The percentage of well‐classified individuals by the four variables taken together may be observed at 89.52%.

### 3.2 Pre‐morbid personality study in AD: Discriminant factor analysis between the AD group informants and the control group informants

Table 4 presents the results of discriminant factor analysis between the AD group informants and the control group informants. As for the pre‐morbid personality characteristics, it is shown that the dimensions openness to experience, conscientiousness, and agreeableness and cluster C are those that best distinguish between the informants, with the AD group informants presenting a lower result in the dimension agreeableness and a higher result in cluster C. It should be noted that the percentage of well‐classified individuals by the four variables taken together is of 80.49%.

The results of the introduction of the 12 personality disorder scales of the PDQ‐4+ are presented in Table 5, in addition to the previous, afore‐mentioned variables, and the object of analysis in Table 4. It is shown that the dimensions openness to experience, schizotypal scale, the dimensions agreeableness and conscientiousness and the obsessive‐compulsive scale are those that best distinguish between the informants, with the AD group informants presenting lower results in the dimension agreeableness and higher results in the dimension conscientiousness and the obsessive‐compulsive scale. It should be noted that the percentage of well‐classified individuals by the five variables taken together is of 86.59%.

## 4 DISCUSSION

In the research of pre‐morbid and current personality variables, which best distinguish AD from the control groups through discriminant factor analysis, the following should be noted.

This study suggests the inclusion of personality evaluation in the diagnosis of AD, based on the information collected from the self‐report format (in interviews) and the following personality variables: The FFM personality dimensions or traits, agreeableness (low) and openness to experience (low), which are the ones that best distinguish AD from the control groups (they presented a percentage of 84.68% of well‐classified individuals by the two variables taken together). It is possible that the screening evaluation of some of the personality disorder scales such as narcissistic, negativistic, dependent, and borderline may add sensitivity to the diagnosis, for which a percentage of 89.52% well‐classified participants in a set of eight variables was observed.

The dimensions Agreeableness (low) and openness to experience (low) are those which best distinguish between the AD group and the control group, with a well‐classified percentage of 84.68%. In accordance with the literature review on this theme and the previously analyzed results, it may be said that these variables appear to be unpredictable. A neuroticism (high) or conscientiousness dimension may have been expected to be the variables with the most discriminant power. However, one should also bear in mind that the studies on dementia within the scope of personality changes are based on parametric or non‐parametric tests and correlations. Nevertheless, the data by Williams et al. (2013) point to the personality trait openness to experience (low), evaluated in a self‐report format, as a pre‐clinical marker of mild cognitive impairment in adults of an advanced age, also in line with authors such as Hoerger et al. (2011) and Suchy et al. (2011). From this perspective, the authors detect early predictors of cognitive impairment, contrasting with the more common views of personality factor conceptualization as risk factors across the life cycle. Duberstein et al. (2010) and Low et al. (2013) underline that the link between low openness to experience and the risk of AD is consistent with recent discoveries on cognitive activity. Moreover, the recent meta‐analyses of Terracciano et al. (2013, 2014) and Wahlin & Byrne (2011), on the above‐mentioned studies, reveal associations, despite their weak effects, between the two dimensions found in the present study, namely agreeableness (low) and openness to experience (low), in the onset of dementia. Authors such as Terracciano et al. (2014) have recently gone as far as to state that the dimensions openness to experience and agreeableness may be protective factors against the expression of AD. Finally, and of equal importance, neuroticism (high) and low agreeableness are also significantly associated with the advanced stages of neurofibrillary tangles in dementia (Terracciano et al., 2013). It should also be noted that low agreeableness emerges as an important marker of psychopathology (e.g., Samuel & Widiger, 2008; Saulsman & Page, 2004, 2005).

This study also suggests the inclusion of personality evaluation in the early diagnosis of AD, in this case, based on the evaluation of Informants, and the following personality variables: The FFM personality traits or dimensions, openness to experience (low), conscientiousness (high) and agreeableness (low), and a high incidence of personality disorders corresponding to cluster C (DSM‐IV—Axis II; PDQ‐4+). These are the variables that best distinguish AD from the control groups, revealing a percentage of 80.49% of well‐classified participants by the four variables taken together. The screening evaluation of some personality disorder scales clearly appear to add sensitivity to the diagnosis (reporting a percentage of 90.24% well‐classified participants), in a set of 10 variables: the scales schizotypal (low), obsessive‐compulsive (high), dependent (high), depressive (low), narcissistic (high), and borderline (high), a high personality disorder global index (PDQ‐4+ Total). Overall, some personality traits or dimensions of the FFM, some personality disorder scales, and cluster C (PDQ‐4+) stand out as useful variables in the discrimination between the retrospective evaluations of the Informants on the possible characteristics of the pre‐morbid personality in AD. Also, there is evidence that AD commonly exhibits changes in personality that occur alongside with mood changes (Wahlin & Byrne, 2011). Investigation of the personality changes, mood alterations, and psychoses in AD may provide insight into the neurobiological bases of these common disorders (Cummings & Victoroff, 1990). It is considered that mood disorders may play a role of confounders in our research.

The interaction effect between a decreased openness and neuroticism to a lifetime depression or recurrent depressive/anxiety symptoms also emerged in literature (Dale et al., 2020). There is evidence of depressive symptoms in dementia related to the premorbid level of openness (Wilson et al., 2008), and that openness was associated with affective symptom evolution in dementia process (Rouch et al., 2019). Furthermore, Caselli et al. (2018) demonstrate that changes in personality, namely an increase in neuroticism and decrease in openness with concomitant subtle changes regarding somatization, depression, anxiety, irritability, and aggression, coincide with the transition from preclinical AD to mild cognitive impairment. It would also be interesting to analyzing the low openness in a long‐term relationship with the other personality dimensions, since despite not having a main role as AD risk factors, openness to experience is thought to have a protective effect of cognitive decline (Duberstein et al., 2010; Williams et al., 2013).

The evidence tends to suggest predisposition and/or pathoplastic relationships between personality and dementia (Segerstrom, 2018). With the disease progression, personality provides a framework to interpret behavioral and psychological symptoms (Sutin et al., 2018). In addition, there are implications of personality research for identifying those at greater risk of AD and the potential of personality‐tailored interventions aimed at the prevention and treatment of AD (Onken & Nielsen, 2019; Terracciano & Sutin, 2019). Certain personality traits are associated with higher risk of AD‐like cognitive impairment, supporting the hypothesis that personality traits can alter the vulnerability and pathoplasticity of disease and therefore modulate related biomarker expression (Zufferey et al., 2017), highlighting intrinsic and mechanistic relations between personality traits and AD pathology (Strikwerda‐Brown, 2022).

Limitations: Lack of use of biomarkers for the diagnosis of Alzheimer's disease; absence of biological data; absence of screening tools for mood disorders; the difficulty of accessing individuals diagnosed with AD at the onset phase; lack of information about duration of the illness, medication or other comorbid diseases; the method of inclusion of the control group sampling may have partially underestimated the occurrence of against personality disorders or maladaptive personality traits; further, personality changes through retrospective assessment by proxies may have introduced some memory bias.

Future studies should be grounded in personality and biological frameworks, addressing, for example, amyloid negative versus amyloid positive individuals, with mild cognitive impairment, matched by age/sex/education/psychiatric illness. It would be useful and important for these data to be replicated or improved in other studies with larger samples and control groups, and the comparison extended to other groups with different dementia etiologies, adding the study of clinical variables of mood disorders. The discriminant analysis could thus offer a helpful contribution toward understanding and delineating the differential diagnosis. Establishing a definition of early diagnosis is equally important, not only in terms of symptomatic content but also of the time gap factor. Perhaps a combination of longitudinal methodology and discriminant analysis in a population sample from middle‐age upward, based on the FFM evaluation in conjunction with clinical and biological measures may enable a further understanding of the period of time prior to the first signs of personality changes in Alzheimer's disease.

## AUTHOR CONTRIBUTIONS

Joana Henriques‐Calado designed the study, wrote the protocol, managed the literature searches, carried out the collection and evaluation of samples, performed data analysis and interpretation, wrote the first draft of the manuscript, and approved the final manuscript.

## CONFLICT OF INTEREST

The author declares no potential conflicts of interest with respect to the research, authorship, and/or publication of this article.

### PEER REVIEW

The peer review history for this article is available at https://publons.com/publon/10.1002/brb3.2938.

## Data Availability

The datasets used and/or analyzed during the current study are available from the corresponding author on reasonable request.
